# No “prevalence bias” in randomized controlled trials on colorectal cancer screening: the importance of clarifying the research question

**DOI:** 10.1007/s10654-025-01303-z

**Published:** 2025-11-12

**Authors:** Ulrike Haug, Mingyang Song, Vanessa Didelez

**Affiliations:** 1 Department of Clinical Epidemiology, Leibniz Institute for Prevention Research and Epidemiology – BIPS, Achterstr 30, 28359 Bremen, Germany; 2 Faculty of Human and Health Sciences, University of Bremen, Bremen, Germany; 3 Departments of Epidemiology and Nutrition, Harvard T.H. Chan School of Public Health, Boston, MA 02115 USA; 4 Clinical and Translational Epidemiology Unit, Massachusetts General Hospital, Harvard Medical School, Boston, MA USA; 5 Division of Gastroenterology, Massachusetts General Hospital, Harvard Medical School, Boston, MA USA; 6 Department of Statistical Methods in Epidemiology, Leibniz Institute for Prevention Research and Epidemiology – BIPS, Bremen, Germany

In October 2022, the first randomized controlled trial (RCT) investigating the effects of population-based colonoscopy screening on colorectal cancer (CRC) incidence and related death at 10 years was published (NordICC trial) [1]. In this pragmatic trial, follow-up data were available for 84,585 participants; 28,220 were in the invited group, 11,843 of whom (42%) underwent screening, and 56,365 were in the usual-care group. In intention-to-treat analyses, the group assigned to screening colonoscopy had a risk reduction of 18% for CRC incidence after 10 years of follow-up, as compared with the usual-care group. Recently published per-protocol analyses using instrumental variable (IV) estimates suggest that colonoscopy screening reduced CRC incidence by 35 to 41% after 10 years [2]. One point of discussion around the NordICC trial was whether it underestimated the effect of colonoscopy given that a certain proportion of the target population had already CRC at baseline, detected by screening colonoscopy [3–6]. According to Brenner et al. a key principle of randomized prevention trials was violated as only “at risk persons who do not yet have the disease one aims to prevent should be included in measures of preventive effects” [3]. The authors called this a “prevalence bias” in the NordICC trial, which would also be relevant to RCTs investigating the preventive effect of flexible sigmoidoscopy. In the meantime, mathematical calculations based on the cumulative incidences provided by RCTs have been conducted estimating the mere preventive effect of flexible sigmoidoscopy and colonoscopy [3, 7, 8] that is supposed to be free of this “prevalence bias”. Also a recent commentary in this journal presented such calculations, in addition to addressing other points about NordICC that will not be discussed here, and called for “more informative metrics of screening efficacy” [9]. To bring more clarity to this discussion, we believe it is important to separate the different research questions underlying the debate and assess them from a decision-making perspective.

In order to speak of bias, it must be clear what the trial aimed to estimate, i.e. from which target value (the estimand) the analysis allegedly deviates. It is therefore key to clarify which of the following different research questions is aimed to be answered.

- **Research question A**: What is the effectiveness of colonoscopy on CRC incidence in persons eligible for screening?
- **Research question B**: What is the effectiveness of colonoscopy on CRC incidence in persons eligible for screening who are *free of CRC at baseline?* This question addresses the mere preventive effect by removing precursors.
- **Research question C**: What is the effectiveness of colonoscopy on CRC incidence in persons eligible for screening who are *free of CRC at baseline* and *who do attend* screening when invited. This question addresses the mere preventive effect by removing precursors among attenders, who may not be representative of the whole (CRC-free) eligible population.

The available RCTs on lower endoscopy answered **research question A**. The approach takes into account that preclinical CRC may already be present when a person undergoes screening, so these CRCs can no longer be prevented. With preventive interventions, one must bear in mind that they may come too late for part of the target population, i.e. their effectiveness may be limited due to this fact, which is relevant when considering the effectiveness of CRC screening on CRC incidence at the population level. We illustrate this with an extreme example: Assume that 4 out of 5 CRC cases that occur per 100 people in their lifetime would already be in the invasive stage at the starting age of screening and only one case would still be in the precursor stage and could be prevented. In this scenario, the incidence of CRC could only be reduced by at most 20% at the population level (perspective of **research question A**). When answering **research question B**, the other 4 CRC cases, where screening comes too late in order to prevent them, would not be considered; this might then suggest that screening can prevent all CRCs, while the precise interpretation is that this is only the case after excluding prevalent cases. In our view, **research question A** is therefore more meaningful to assess the impact that screening can have on CRC incidence at the population level. From a public health perspective, it is important to take into account that not all CRC cases that are preventable by endoscopy can be prevented unless screening were to start from birth onwards, i.e. **research question A** is the most relevant one for public health decision-makers.

Which of the research questions is most relevant for the individual person who has to decide whether or not to participate in screening? Since the person does not yet know at this point whether or not he or she already has CRC, an effect estimate that refers to persons who are free of CRC (perspective of **research questions B and C**), i.e. that conditions on being free of CRC, is not the relevant one at the time when the decision has to be made. Moreover, a person who has to decide for or against screening cannot know if he or she is similar to attenders in an RCT (perspective of **research question C**). So, also for individuals, **research question A** is the relevant one to support decision making. For persons in whom precursors were detected and removed at screening, it might be interesting to which extent screening had decreased their CRC risk, but this question arises *after* having undergone screening with removal of precursors, so *after* they have made their decision regarding screening participation. The next decision persons with removed precursors have to make concerns undergoing surveillance colonoscopy, but evidence supporting decision making about surveillance would require yet another study design and is unrelated to the above research questions.

At this point, it can be summarized that for both public health decision-makers and for individuals, **research question A** is the relevant one to support decision making. The only difference between the perspective of the decision maker and the individual person is that for the individual person, the per-protocol effect on **research question A** is typically the most relevant, while for decision makers, also (or mainly) the intention-to-screen effect is of interest. Accordingly, the available RCTs on lower endoscopy answer the relevant research question and were designed for this purpose, i.e. there was no so-called “prevalence bias”.

Undoubtedly the answer to **research questions B and C** would also be interesting, mainly from a scientific point of view. However, can they be answered? To clarify this, it is useful to imagine the design of a hypothetical RCT that would answer **research question B** (Fig. 1): Before randomization, all persons would undergo a first colonoscopy, but without removal of precursors. Those with CRC detected at this colonoscopy would be excluded from the trial. In a next step, the remaining individuals (all free of CRC) would be randomized to the intervention or the control arm. Persons randomized to the intervention arm would undergo another colonoscopy, now with the removal of precursors. Persons in the control arm would not undergo another colonoscopy, i.e. precursors detected at first colonoscopy would remain and may develop into CRC. In both groups, CRC incidence during follow-up would be assessed and compared. Although it is clear that such a trial would be unethical due to the non-removal of detected precursors in those randomized to the control arm, formulating such a hypothetical trial helps to illustrate the study-design and data that would be needed to answer **research question B**. (Note that we may extend the hypothetical design to address **research question C** by excluding all those who refuse to attend a screening colonoscopy.) Observational data to emulate such a trial do not exist because in the real-world setting, CRC detection and removal of precursors is done at the same time. Any attempt to answer **research question B** with observational data needs to rely on further unverifiable assumptions (in addition to assuming that we can adjust for any confounding) due to the fact that both precursors AND preclinical cancers are removed in the exposed (screening) group but not in the unexposed (control) group. So not only the prevalence of precursors differs between groups after screening, but there is also an imbalance in the prevalence of preclinical cancer. Existing observational studies—except for those designed based on target trial emulation [10–13]—may have aimed to estimate the mere preventive effect of polypectomy on CRC incidence, although they typically did not explicitly state this. The underlying designs varied but had in common that there was a misalignment at time zero. This led to non-consideration of screen-detected (i.e. preclinical) CRCs in the exposed (screening) group, which inevitably biases the comparison to the unexposed (control) group, as demonstrated by Braitmaier et al. [13]. Even if polypectomy had no effect on CRC incidence, CRC incidence during follow-up would be lower in the screened group when using such a design because persons with preclinical CRCs are removed in the screening group but not in the control group [13]. Other attempts to estimate the mere preventive effect of polypectomy on CRC incidence, e.g. by calculations based on the cumulative incidences provided by RCTs, require further unverifiable assumptions or – as is the case for calculations presented recently [9] – are only applicable to “attenders” (**research question C**) where extrapolation to non-attenders and those still making the decision in the future is problematic.^1^

In conclusion, the available RCTs investigating the preventive effect of lower endoscopy screening on CRC incidence answered the research question that is arguably most relevant to decision making (**research question A**). They were designed for this purpose, i.e. there was no “prevalence bias”. The research questions regarding the effectiveness of colonoscopy on CRC incidence in persons free of CRC at baseline (**research questions B and C**), corresponding to the mere preventive effect by removing precursors, are mainly interesting from a scientific perspective. RCT designs that would answer these questions are unethical, observational data cannot provide a valid answer and mathematical attempts to answer them require a number of assumptions or are only applicable to ”attenders”, i.e. who already made their decision.

Generally, we call for (i) a clear and explicit separation of the different research questions that may be of interest, especially regarding the desired subpopulation to which the effect applies, and (ii) a clear and explicit separation of issues of bias from the fact that studies may address different research questions.

**Fig. 1:**
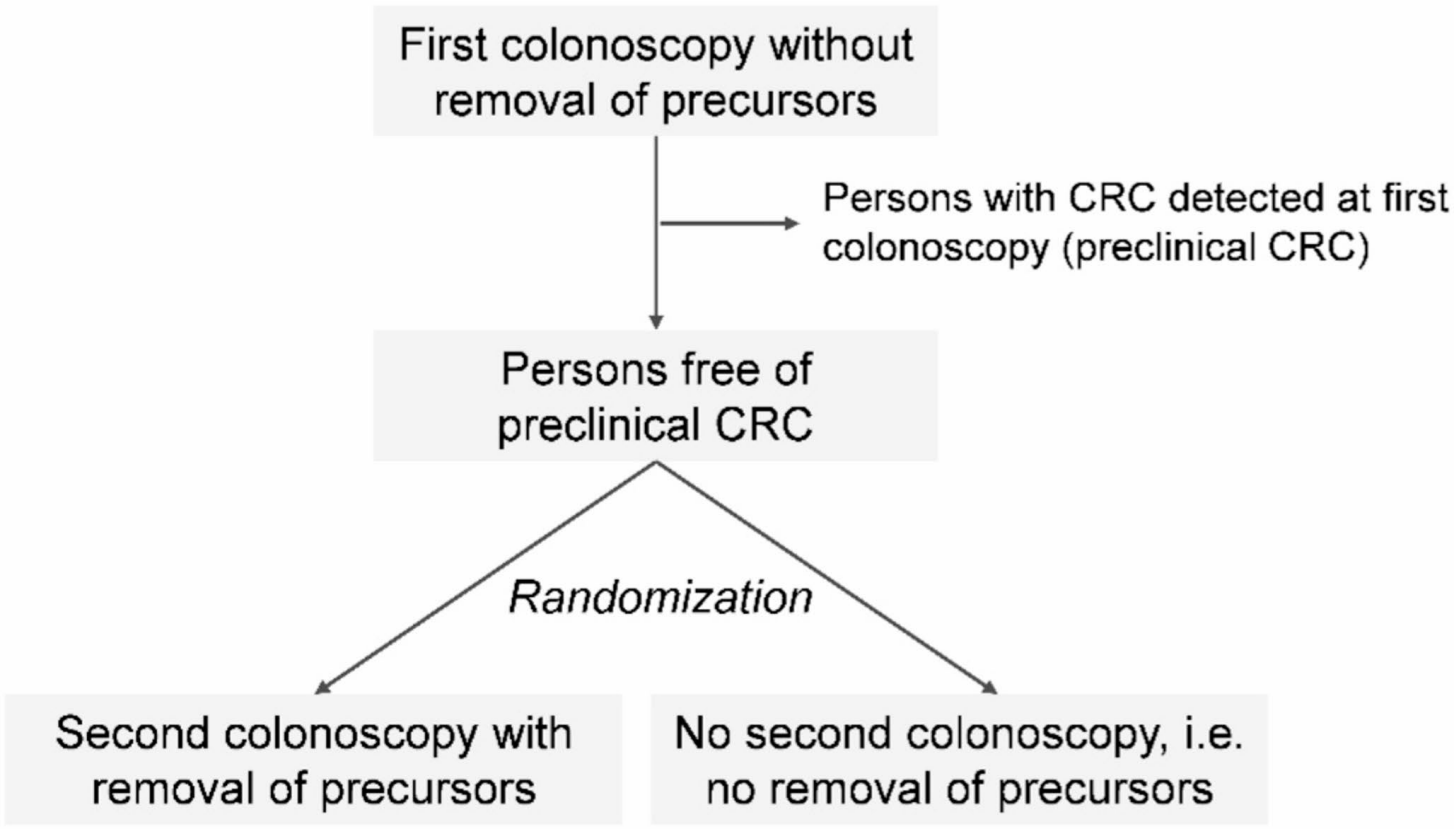
Illustration of a hypothetical RCT designed to answer research question B, i.e. the effectiveness of colonoscopy on CRC incidence in individuals who are free of CRC at baseline, corresponding to the mere effectiveness of removing precursors

## Footnotes

1 The calculations presented in [9] aiming to estimate the preventive effect of polypectomy on the screeners (or attenders) are essentially (and implicitly) exploiting the randomization as an instrumental variable and are similar in spirit to one of the three analysis approaches of Shi et al. [2] with the additional distinction between early detected and incident cases, which relies on further assumptions.
